# Sustainable production of battery-grade nickel via hydrogen reduction of saprolite

**DOI:** 10.1038/s41598-026-36516-z

**Published:** 2026-01-16

**Authors:** Taejun Park, Seongsoo Han, Wonjae Lee, Joobeom Seo, Kimin Roh

**Affiliations:** 1 Resources Utilization Division, Korea Institute of Geoscience & Mineral Resources (KIGAM), 124 Gwahak-ro, Yuseong-gu, Daejeon, 34132 Republic of Korea; 2 Resource Engineering, University of Science Technology (UST), 217 Gajeong-ro, Yuseong- gu, Daejeon, 34113 Republic of Korea

**Keywords:** Nickel, Laterite, Hydrogen reduction, Saprolite, Nickel pig iron, Chemistry, Energy science and technology, Engineering, Environmental sciences, Materials science

## Abstract

Nickel production from saprolite—a major laterite source—is critical for the electric vehicle battery supply chain but is currently constrained by the high carbon footprint of the conventional Rotary Kiln-Electric Furnace (RKEF) process. Hydrogen-based reduction offers a sustainable alternative; however, optimizing the reaction kinetics and phase separation efficiency remains a challenge for industrial application. In this study, we investigated the hydrogen reduction behavior of saprolite ore using a dynamic reduction system to maximize Nickel Pig Iron (NPI) recovery. The effects of reduction time, temperature, gas flow rate, and particle size were systematically evaluated. The results revealed that particle size is the governing factor overcoming the diffusion resistance within the Mg-rich silicate matrix. Optimal reduction efficiency (~ 20 wt% mass loss) was achieved rapidly within 15 min at 900 °C with a particle size of -45 μm. Furthermore, a high-grade NPI (Fe ~ 73 wt%, Ni ~ 25 wt%) was successfully produced with a clear separation from the silicate slag phase. These findings demonstrate that controlling physical parameters based on mineralogical constraints is key to enhancing the reduction efficiency of hydrogen reduction, providing a viable pathway for low-carbon nickel smelting processes.

## Introduction

Nickel is a critical strategic metal essential to various high-tech industries, including national defense, aerospace, and next-generation energy-storage systems^1,2^. Its demand has surged rapidly in recent years, particularly owing to the accelerated growth of the electric-vehicle market^3^. Notably, nickel-based materials account for nearly 8% of the total carbon footprint in Li-battery production, underscoring the urgent need to address the environmental implications of nickel usage in batteries^4^. Conventional nickel production processes emit a significant amount of carbon dioxide (CO₂), raising serious environmental concerns and prompting the need for more sustainable process technologies^5^.

Laterite-type nickel ores, which serve as the primary raw material for nickel production, are broadly categorized into limonite and saprolite types. Among them, saprolite ores consist predominantly of Mg-rich silicate minerals and typically contain a relatively high nickel content of > 1.5%^6–9^. Saprolite nickel ore exists as silicate minerals containing Mg and Fe, with nickel structurally bound within the mineral lattice through isomorphic substitution. The processing of nickel laterites is generally categorized into two routes based on the ore composition. Hydrometallurgical methods, such as High-Pressure Acid Leaching (HPAL), are predominantly used for low-grade limonite ores (high Fe, low Mg). In contrast, pyrometallurgical processes, particularly the Rotary Kiln–Electric Furnace (RKEF) route, remain the primary technology for processing saprolite ores. This is because the high magnesium content in saprolite causes excessive acid consumption in hydrometallurgical leaching, making pyrometallurgy the preferred method for this ore type. Currently, saprolite nickel ores are primarily processed using the rotary kiln–electric furnace (RKEF) route, which accounts for approximately 95% of global saprolite-based nickel production^10^. The RKEF process employs coal as both a fuel and reducing agent, resulting in substantial CO₂ emissions. Depending on the ore grade and energy source, the carbon intensity typically ranges from 30 to 45 t-CO_2_/t-Ni, but can reach over 60 t-CO_2_/t-Ni in Nickel pig iron (NPI) production chains heavily reliant on coal-fired electricity^5,11^. Although alternative processes such as NPI-to-matte conversion and high-pressure acid leaching (HPAL) have recently attracted attention, these methods are known to emit 3–4 times more CO₂ than conventional routes^12^. Furthermore, increasing environmental pollution and social conflicts related to nickel production have intensified ESG-related concerns, reinforcing the urgent demand for more ecofriendly and sustainable nickel smelting technologies^13^.

Accordingly, the development of low-carbon nickel smelting technologies is imperative, and hydrogen-based reduction processes have attracted attention as a promising alternative. Hydrogen-based reduction technology can significantly reduce greenhouse-gas emissions compared to conventional coal-based processes and is considered a viable solution to support global efforts toward carbon neutrality. Numerous researchers have investigated the reduction of nickel oxides using hydrogen gas, taking into account various reaction parameters, such as temperature, reaction time, gas composition, gas flow rate, and pressure. Oliveira et al.^14^ investigated the kinetic behavior of hydrogen reduction of limonitic nickel ore using a rotary kiln. Experiments were performed over the temperature range of 400–800 °C using pure hydrogen gas at a flow rate of 10 L/min and a kiln rotation speed of 20 rpm. The results indicated that hematite was reduced to magnetite in the range of 400–550 °C and that further reduction to metallic Fe occurred at temperatures between 550 and 800 °C. The apparent activation energies for the reduction reactions were reported as 46.2 kJ/mol for hematite to magnetite and 29.5 kJ/mol for magnetite to metallic Fe. Liu et al.^15^ performed hydrogen reduction experiments on lateritic nickel ore at temperatures ranging from 600 to 1100 °C, comparing conditions with and without up to 25 wt% addition of Na₂S₂O₃. A H₂–N₂ gas mixture with a total flow rate of 27 L/min was used, and the hydrogen concentration was varied from 20% to 70%. The reduced products were crushed and separated using a magnetic separator, with the optimal hydrogen concentration reported as 45%. In a related study, Wijenayake et al.^16^ reduced limonitic nickel ore at 650 °C for 1 h using hydrogen gas, followed by a two-step hydrometallurgical process (leaching and cementation) to produce a ferronickel precipitate with a nickel content of approximately 10%. Satritama et al.^11^ conducted a systematic experimental study on the hydrogen–argon reduction of saprolite nickel ore. The effects of the temperature, gas composition, and reaction time on the reduction behavior were investigated over the temperature range of 500–900 ℃, with hydrogen concentrations of 25% and 75% and reaction durations ranging from 15 min to 3 h. Phase analysis of the reduction products revealed the formation of olivine, pyroxene, iron–silicate, spinel, metallic Fe, and ferronickel phases. The results demonstrated that higher temperatures led to an increase in both the extent of reduction and the formation of metallic phases.

However, studies on the hydrogen reduction of saprolite nickel ore remain insufficient for industrial implementation. Most previous investigations have been confined to static, small-scale experiments, which fail to represent the dynamic gas-solid contact conditions of a rotary kiln. Furthermore, the correlation between the ore’s physicochemical properties and the reduction efficiency—essential for process optimization—has not been fully elucidated.

Therefore, this study aims to develop an optimized hydrogen reduction process for clean NPI production using a dynamic rotary reactor. We systematically assessed the effects of critical engineering parameters, including temperature, gas flow rate, and particle size, on the reduction/mass loss (mass loss, wt%) and metallic phase growth. By elucidating the mechanism of phase transformation and slag separation, this work provides essential engineering data for scaling up the eco-friendly hydrogen-based smelting technology.

## Experimental section

### Sample preparation

The saprolite ore was sourced from New Caledonia and provided by SNNC Co., Ltd., Korea. The sample was wet-sieved without prior crushing or grinding using a vibratory sieve shaker (Analysette 3 Pro, Fritsch, Germany) and was classified into four particle size fractions: +355 μm, + 125–355 μm, + 45–125 μm, and − 45 μm. In total, four saprolite ore samples were prepared for further experiments and were referred to as + 355 μm, + 125–355 μm, + 45–125 μm, and − 45 μm.

### Mineralogy

To analyze the mineralogical characteristics of the prepared saprolite samples, quantitative X-ray diffraction (QXRD, Philips X’Pert MPD, PANalytical X’Pert3 Powder, Malvern Panalytical, Netherlands) and scanning electron microscopy with energy-dispersive X-ray spectroscopy (SEM-EDS; TM3000–SwiftED 3000, Hitachi, Japan) were employed. In addition, the chemical composition of the ore was determined using X-ray fluorescence (XRF; MXF-2400, Shimadzu, Japan) and inductively coupled plasma–atomic emission spectrometry (ICP-AES; 5300 DV, PerkinElmer, USA). The loss on ignition (LOI) for the XRF analysis was determined by measuring the mass loss of the samples after heat treatment at temperatures above 995 °C in a muffle box furnace (Ajeon Heating Industrial Co., Ltd.).

### Hydrogen reduction process

**Fig. 1:**
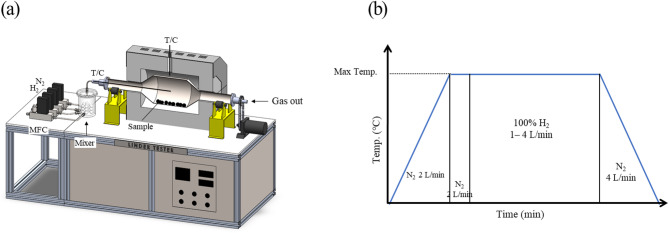
(**a**) Schematic of the dynamic reduction experimental apparatus. (**b**) Heat and reduction-gas injection pattern for reduction tests.

The reduction experiments of saprolite nickel ore were conducted using a dynamic reduction system designed to simulate the rotary kiln process. The configuration of the apparatus and the thermal treatment pattern are illustrated in Fig. 1. This system was specifically engineered to enable gas–solid reactions within a rotating reaction chamber under controlled temperature and gas flow conditions. The reaction chamber was made of stainless steel 310. It was 1 m long and was operated at a rotation speed of 2 rpm. The internal temperature of the chamber was continuously monitored in real time using a precisely calibrated R-type thermocouple. A reducing atmosphere was established using high-purity hydrogen (99.995%) and high-purity nitrogen (99.995%) gases. The hydrogen flow rate was precisely controlled using a mass flow controller (MFC) (Fig. 1(a)). The total gas flow was maintained within the range of 1–4 L/min. To ensure safety, hydrogen leak sensors were installed around the apparatus to detect any gas leakage in real time. Reduction experiments were performed under four temperature conditions: 800, 850, 900, and 950 ℃. The reaction time was varied from 15 to 120 min to evaluate the time-dependent reduction behavior. For each test, 50 g of saprolite nickel ore in powder form was loaded into the center of the reactor. Prior to the reaction, high-purity nitrogen gas was introduced to establish an inert atmosphere inside the chamber, and the system was heated to the target temperature at a ramp rate of 10 ℃/min. When the desired temperature was reached, the nitrogen gas was switched to hydrogen to initiate the reduction. After the reaction, the hydrogen was purged with nitrogen again, and the system was cooled naturally to room temperature (Fig. 1(b)). The extent of reduction was quantitatively evaluated by measuring the weight change of the sample after the experiment. In addition, XRD analysis was performed to examine the phase composition and structure of the reduced products.

### Melting process for NPI production

**Fig. 2:**
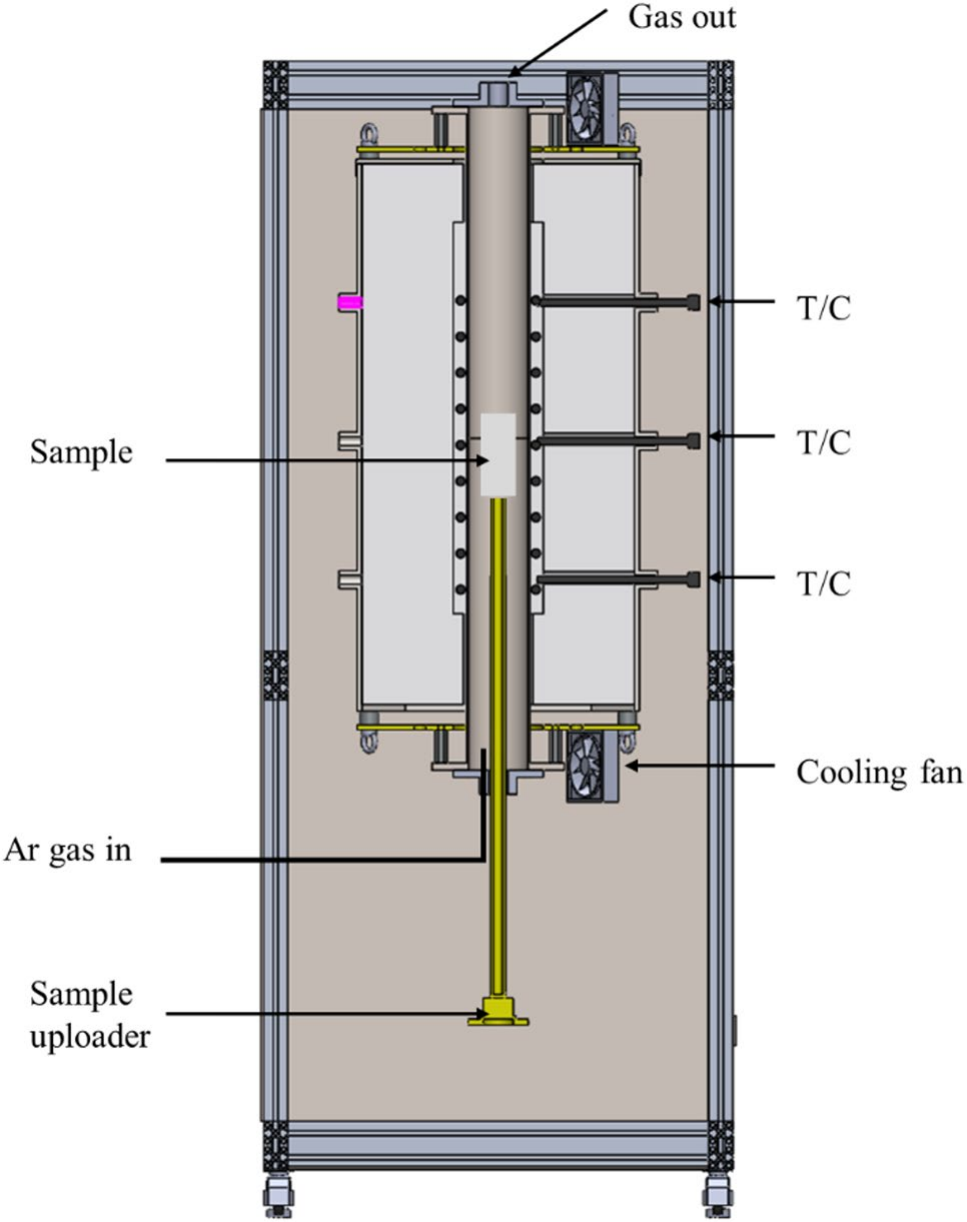
Schematic of the vertical tube furnace.

To simulate the electric-furnace process for producing NPI from hydrogen-reduced nickel ore, melting experiments were conducted in a vertical tube furnace. The experimental setup of the vertical tube furnace is shown in Fig. 2. The temperature profile within the alumina tube was monitored using a precisely calibrated B-type thermocouple, and temperature stability was maintained by employing a three-zone heating system. An inert atmosphere was ensured throughout the melting process via a continuous Ar gas flow of 2 L/min. Exhaust gases were safely removed via a gas trapper and exhaust duct. To accommodate larger sample quantities, the hydrogen-reduced nickel oxide powder was compacted into pellets without the addition of any binders or secondary reductants to prevent impurity contamination, and then loaded in layers into a magnesia crucible, with a total mass of approximately 500 g melted per run. The molten sample was held at 1550 ℃ for 2 h to achieve homogenization. Natural cooling was conducted inside the vertical tube furnace under the continuous Ar flow to prevent oxidation. The NPI produced after the melting process was analyzed using ICP-AES and field-emission electron probe microanalysis (FE-EPMA; JXA-IHP200F, JEOL Ltd., Japan).

## Results and discussion

### Mineralogy

**Fig. 3:**
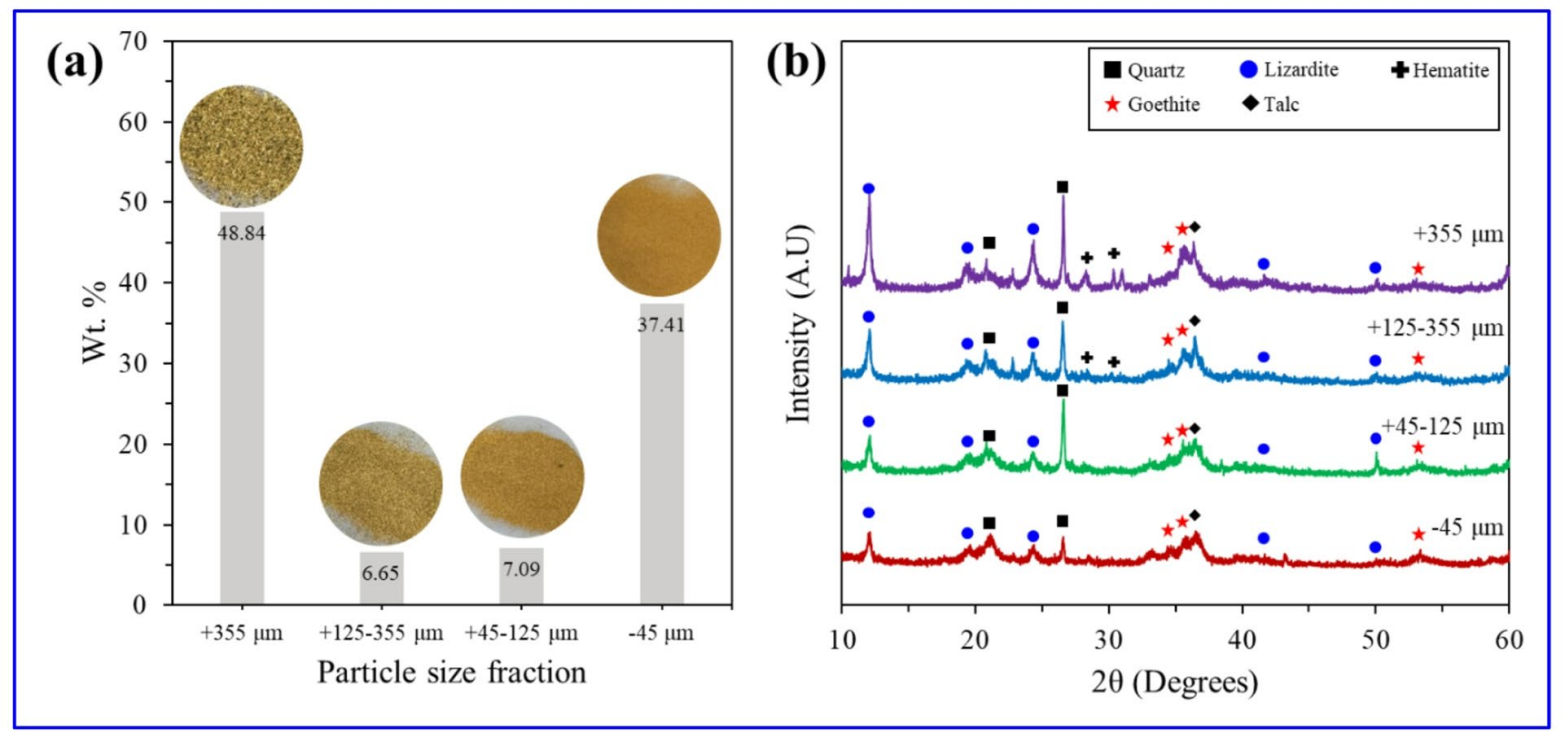
Particle size distribution and XRD patterns of saprolite ore samples. (**a**) Photographs and weight distribution of various particle size fractions obtained via wet sieving: +355 μm, + 125–355 μm, + 45–125 μm, and − 45 μm. (**b**) XRD pattern for each size fraction.

Figure 3 shows the particle size distribution and XRD patterns of the saprolite ore samples by size fraction and Table 1 presents the corresponding QXRD results. As shown in Fig. 3(a), the + 355 μm and − 45 μm fractions account for > 85% of the total sample, indicating a heterogeneous size distribution with large proportions of both coarse and fine particles. The sample images show that the coarse particles exhibit a relatively light (whitish) color, while the ore becomes increasingly reddish as the particle size decreases. In Fig. 3(b) and Table 1, XRD analysis by particle size fraction confirms distinct differences in mineral phase distribution depending on particle size. Overall, prominent peaks of goethite (★, red stars) and lizardite (●, blue circles)—a serpentine-group mineral—were identified, which are typical of laterite ores. Notably, the coarse fraction (+ 355 μm, + 125–355 μm) exhibited stronger lizardite peaks, while the fine fraction (− 45 μm) exhibited an increased relative intensity of goethite peaks. These results suggest that serpentine minerals are relatively concentrated in the coarse particles, whereas goethite is enriched in the finer particles. Such mineralogical distribution trends are commonly observed in laterite ores and have been previously reported^17,18^. This size-dependent distribution reflects the contrasting mineralogical origins and weathering behaviors of these phases: lizardite is a residual serpentine mineral that retains relatively coarse textures during saprolitization, whereas goethite is a secondary weathering product formed by intense lateritization and typically occurs as fine-grained aggregates that preferentially report to the finer fractions^19^.

Table 1 presents the chemical composition and metal content of the saprolite samples classified by particle size fraction. According to the XRF analysis, the coarse fraction (+ 355 μm) exhibited high contents of SiO₂ (42.81 wt%) and MgO (26.53 wt%), while the Fe₂O₃ content was relatively low (14.54 wt%). As the particle size decreased, the Fe₂O₃ content increased, whereas the SiO_2_ and MgO contents decreased. This indicates that Fe-bearing minerals, particularly goethite, are concentrated in the finer fraction, which is consistent with the aforementioned XRD results. The ICP-AES analysis of Fe and Mg revealed similar trends, suggesting the enrichment of Fe-rich mineral phases in the fine particles. Therefore, it is expected that the finer fraction will exhibit higher reduction reactivity and metal recovery efficiency in the hydrogen reduction process. Meanwhile, in the coarse fraction, where serpentine minerals with high SiO_2_ and MgO contents are dominant, the reduction efficiency is anticipated to be relatively limited. These findings suggest that for optimizing the subsequent nickel recovery process, classification and adjustment of reduction conditions based on particle size-dependent chemical and mineralogical differences should be considered.

**Fig. 4:**
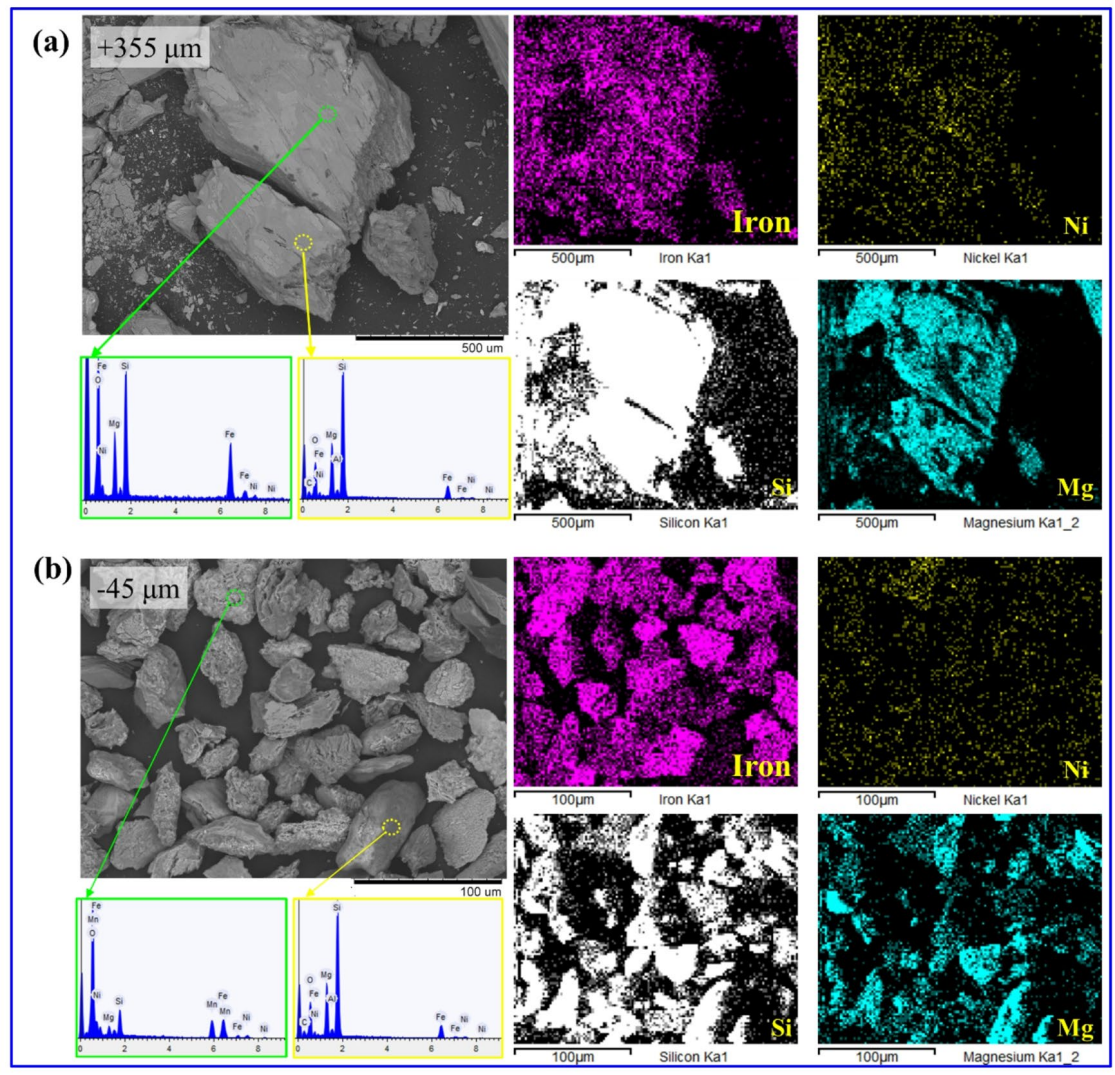
SEM-EDS analysis with elemental mapping images (Fe, Ni, Mg, and Si) of saprolite ore samples: (**a**) + 355 μm fraction; (**b**) − 45 μm fraction.

Figure 4 presents the SEM-EDS analysis results and elemental mapping images (Fe, Ni, Mg, and Si) for the + 355 μm (Fig. 4(a)) and − 45 μm fractions (Fig. 4(b)) of the saprolite ore samples. In both size fractions, Ni was observed to be uniformly distributed across various mineral phases without being concentrated in specific minerals or localized areas. Elemental mapping confirmed that Ni was generally dispersed along with Fe- and Mg-bearing minerals, indicating that it likely exists in a lattice-substituted form within goethite and serpentine minerals. Therefore, applying a physical separation process to selectively separate serpentine and goethite is considered neither technically feasible nor economically viable. Instead, it is judged that the most appropriate processing strategy is to directly feed all samples into the hydrogen reduction process without prior physical separation. In summary, the SEM-EDS results indicated that nickel was uniformly distributed within both serpentine and goethite minerals, rather than being concentrated in specific phases. Complementary mineralogical analyses (XRD, XRF, and ICP-AES) confirmed that serpentine was more abundant in the coarse fraction, whereas goethite was enriched in the fine fraction. Accordingly, particle size-dependent variations in the Fe, Mg, and Si contents are expected to influence the reduction efficiency.

**Table 1:** Quantitative Xrd analysis of mineralogical composition by particle size fraction (t.r. Indicates trace amounts below the quantitative detection limit).

Sample	XRF (wt%)											ICP-AES (wt%)						
	SiO_2_	Al_2_O_3_	Fe_2_O_3_	CaO	MgO	K_2_O	Na_2_O	TiO_2_	MnO	*P*_2_O_5_	Ig.loss	Ni	Cr	Mn	Al	Mg	Ca	Fe
+ 355 μm	42.81	2.05	14.54	0.66	26.53	0.03	0.19	0.09	0.50	0.01	9.52	0.70	0.42	0.17	0.63	8.04	0.25	4.76
+ 125–355 μm	41.02	2.35	21.58	0.55	20.96	0.08	0.22	0.09	0.70	0.01	8.85	0.77	0.5	0.25	0.68	5.67	0.19	7.19
+ 45–125 μm	39.46	2.21	27.53	0.52	16.52	0.04	0.12	0.09	0.59	0.01	9.42	0.86	0.48	0.21	0.68	4.86	0.20	9.16
–45 μm	32.23	2.26	35.59	0.33	14.63	0.04	0.13	0.11	0.39	0.02	10.91	0.97	0.28	0.14	0.66	4.45	0.13	11.8

### Hydrogen reduction

**Fig. 5:**
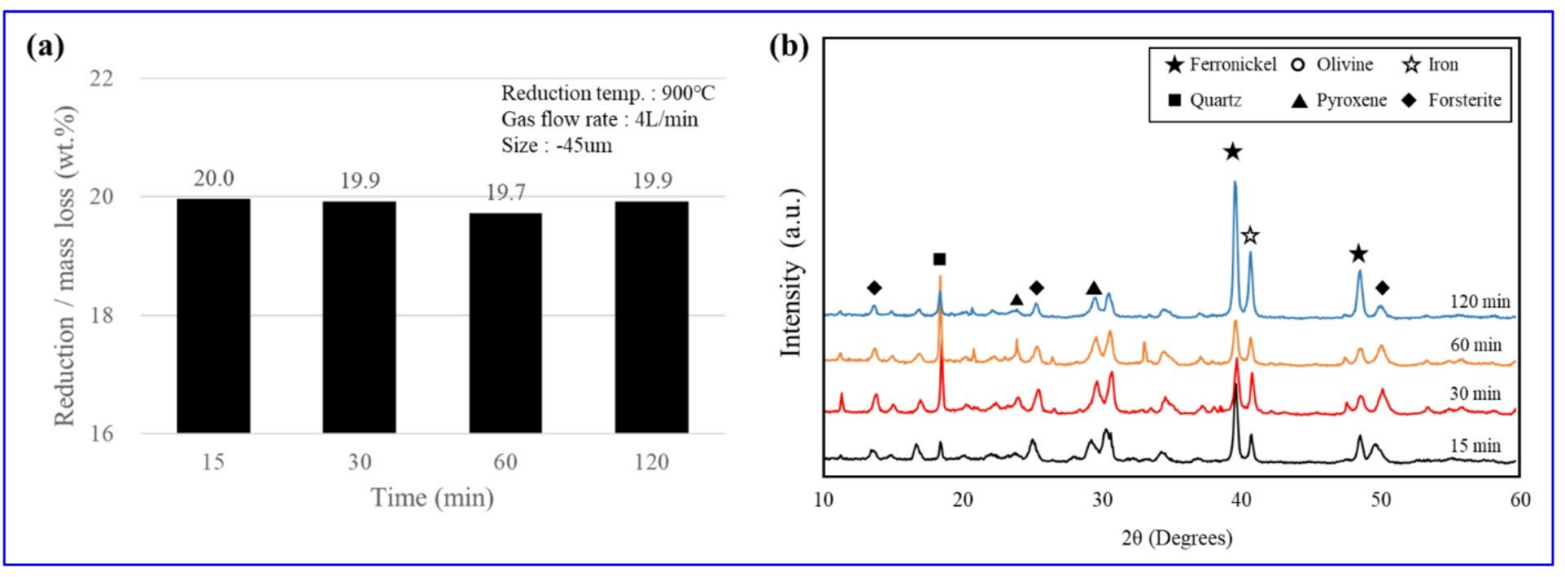
Reduction/mass loss and phase change according to hydrogen reduction time: (**a**) reduction rate; (**b**) XRD peaks.

At 900 ℃ under a hydrogen atmosphere (3 L/min) with a feed size of − 45 μm, the change in reduction extent (mass loss, wt%) over time is shown in Fig. 5(a). It should be noted that the ‘mass loss’ reported in this study serves as a comprehensive indicator of the total thermochemical transformation, encompassing both the thermal dehydroxylation of hydrated minerals (e.g., lizardite, goethite) and the removal of oxygen during the hydrogen reduction of metal oxides. As elucidated by Manzoor et al.^5^. and Satritama et al.^11^., the total weight change at high temperatures represents the cumulative result of these overlapping reactions. Given that the observed mass loss (~ 20.0 wt%) closely matches the theoretical sum of the ore’s loss on ignition (LOI) and the oxygen content associated with Fe/Ni metallization, this metric effectively characterizes the completion of the overall reduction process. The reduction/mass loss (mass loss, wt%) reached 20 wt% at 15 min and remained essentially unchanged over 30–120 min, indicating negligible time dependence. The mass loss reached a stable plateau of ~ 20.0 wt% within 15 min, and subsequent variations over the 30–120 min range were negligible. This confirms that the thermochemical reactions reached completion rapidly within the initial 15 min. Figure 5(b) presents the XRD patterns according to reduction time. Peaks assigned to olivine (○), pyroxene (▲), Forsterite (◆), and quartz (■) were already evident at 15 min, confirming the formation of Mg-rich silicates (Mg₂SiO₄) via serpentine decomposition. Over time, the peaks of metallic Fe (☆) and ferronickel (★) intensified significantly in the 60–120 min range. This behavior is attributed to growth/coarsening and improved crystallinity of metallic phases rather than a further increase in the overall reduction/mass loss (mass loss, wt%). These results demonstrate that hydrogen reduction of the nickel oxide ore at 900 °C proceeds rapidly and that extending the reaction time exerts only a minor influence on the reduction rate.

**Fig. 6:**
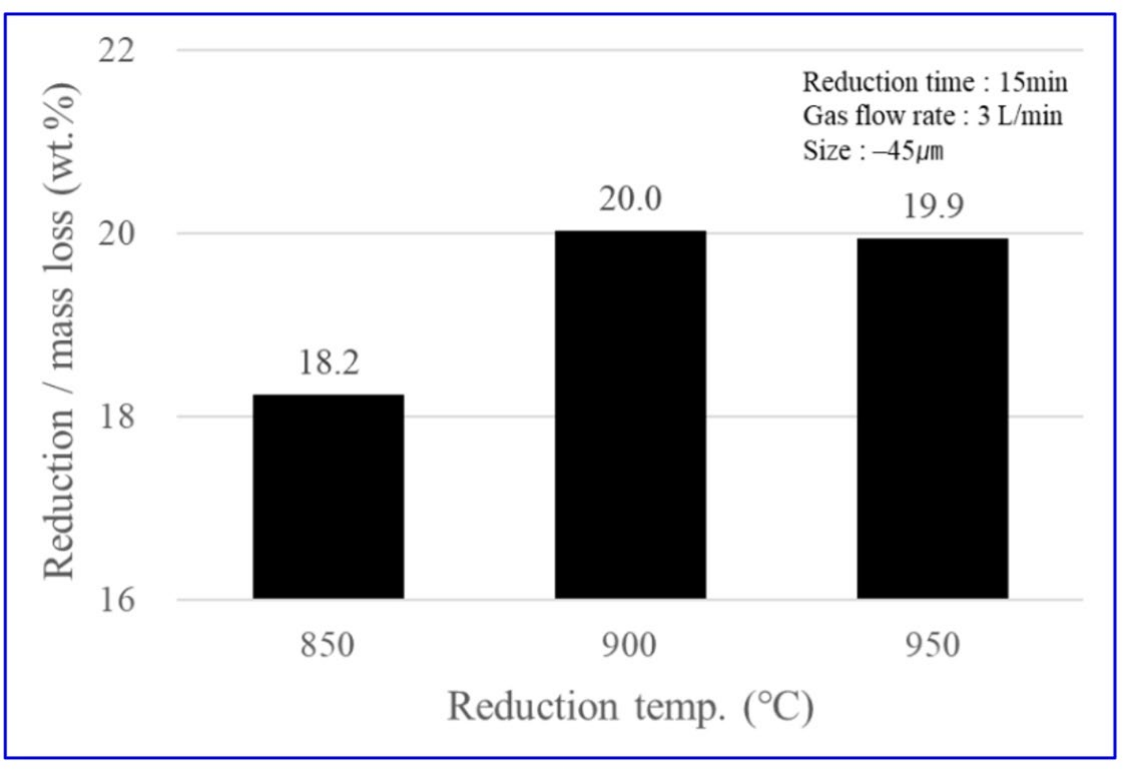
Reduction/mass loss according to reduction temperature.

Figure 6 shows the temperature dependence of the reduction/mass loss (mass loss, wt%) at a fixed reduction time of 15 min under a hydrogen atmosphere (3 L/min) with a feed size of − 45 μm. The reduction/mass loss (mass loss, wt%) increased from 18.2 wt% at 850 ℃ to 20.0 wt% at 900 ℃ and approached a plateau at 19.9 wt% at 950 ℃. These results indicate that a temperature of 900 ℃ is sufficient to achieve near-complete reduction within the short residence time of 15 min. The reaction sequence involved in the hydrogen reduction of nickel oxide ore has been extensively validated in previous studies.

Satritama et al.^11^ reported that reducing saprolite nickel ore at 500 °C using an H₂–Ar gas mixture containing 25% H₂ led to the formation of metallic Fe via the reduction of goethite and hematite, indicating a relatively substantial extent of reduction. The overall reaction can be expressed as follows:

1 $$FeO\left( {OH} \right)\left( {goethite} \right) + {\text{ }}3/2{H_2} \to Fe{\text{ }}\left( {metal} \right) + 2{H_2}O$$

2 $$F{e_2}{O_3}\left( {hematite} \right) + 3{H_2} \to Fe\left( {metal} \right) + {\text{ }}3{H_2}O$$

Serpentine remained stable at 500 °C but began to decompose at temperatures above 600 °C. The decomposition of serpentine into various magnesium silicates at ≥ 600 °C was proposed by Brindley and Hayami^20^ and later confirmed experimentally by Chen et al.^21^. Serpentine first decomposes to olivine, and subsequent reactions between olivine and quartz form pyroxene. The decomposition reactions of serpentine can be expressed as follows:

3 $$2{\left( {M{g_{1 - x - y}},{\text{ }}F{e_x},{\text{ }}N{i_y}} \right)_3}S{i_2}{O_5}\left( {OH} \right) \to 3{\left( {M{g_{1 - x - y}},{\text{ }}F{e_x},{\text{ }}N{i_y}} \right)_2}Si{O_4}\left( {olivine} \right) + Si{O_2}\left( {quartz} \right) + 4{H_2}O$$

4 $${\left( {M{g_{1 - x - y}},{\text{ }}F{e_x},{\text{ }}N{i_y}} \right)_2}Si{O_4}\left( {olivine} \right){\text{ }} + {\text{ }}Si{O_2}\left( {quartz} \right) \to 2\left( {M{g_{1 - x - y}},{\text{ }}F{e_x},{\text{ }}N{i_y}} \right)Si{O_3}\left( {pyroxene} \right).$$

Satritama^11^ and Zhang et al.^22^ found olivine, pyroxene, and Fe_x_Ni_y_ (metal) in solid reduction products at temperatures above 600 °C. They confirmed that Fe_x_Ni_y_ (metal) was produced by the reduction of serpentine minerals, i.e., olivine and pyroxene, and showed that this occurred according to the following simplified reaction equation:

Here, V refers to metal vacancies in olivine or pyroxene.

In the reduction process of the above nickel oxide ore, the Fe_x_Ni_y_ (metal) phase is separated from the olivine and pyroxene phases at a reduction temperature of 600 °C or higher. However, this is the result of sufficient reduction time, and a temperature of 900 °C or higher is needed to complete the reduction within a short time (15 min) using pure hydrogen, as in this study.

**Fig. 7:**
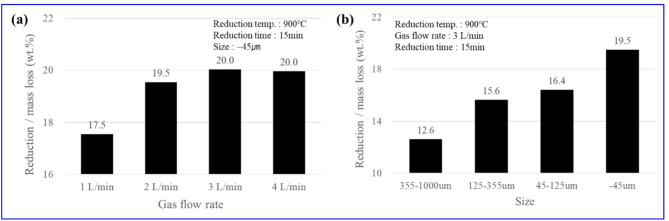
Reduction/mass loss changes according to reduction conditions: (**a**) gas flow rate; (**b**) particle size.

Figure 7 shows the effects of the reduction-gas flow rate and nickel oxide particle size on the Reduction/mass loss at a fixed reduction temperature of 900 °C and reduction time of 15 min. When the particle size was fixed at − 45 μm (Fig. 7(a)), the Reduction/mass loss was measured by gradually increasing the gas flow rate from 1 to 4 L/min. The results indicated that the Reduction/mass loss started at 17.5 wt% at 1 L/min, increased to 19.5 wt% at 2 L/min, and reached a saturation stage of 20.0 wt% at both 3 and 4 L/min. This trend suggests that in the low-flow rate region (> 3 L/min), boundary-layer reduction of the feedstock dominates, but at ≥ 3 L/min, sufficient hydrogen supply enables chemical reactions and diffusion within the particles. This aligns with previous results indicating a gradual increase in Reduction/mass loss within the 900–950 °C range. From a process optimization perspective, a flow rate of ≥ 3 L/min is proposed as the practical critical flow rate. While the reduction-gas flow rate was maintained at 3 L/min, the particle size of the nickel oxide ore was reduced in the sequence of 355–1000 μm, 125–355 μm, 45–125 μm, and − 45 μm (Fig. 7(b)), and the Reduction/mass loss was found to increase distinctly to 12.6, 15.6, 16.4, and 19.5 wt%, respectively. This is attributed to the increased surface area and reduced diffusion distance within the particles, which facilitated the formation of reaction interfaces, and indicates a reduction in internal diffusion resistance within the Mg-rich silicate matrix formed after serpentine decomposition. These microstructural observations align with the XRD analysis conducted on samples from varying temperatures and particle sizes, which confirmed that the constituent phases remained consistent with those observed in Fig. 5(b). This consistency indicates that while parameters such as particle size and gas flow rate significantly enhance the reduction speed by reducing internal diffusion resistance, they do not alter the fundamental phase transformation pathway (i.e., decomposition of serpentine into forsterite/enstatite and metallization). Therefore, the limited increase in the overall reduction rate, despite the strengthening of metallic Fe and ferronickel peaks over time, is attributed to the physical coalescence of metallized particles rather than the formation of new mineral phases. In summary, practical hydrogen reduction conditions for nickel oxide ore were derived, under which a high Reduction/mass loss of approximately 20 wt% could be reproducibly achieved at 900 ℃, 15 min, gas flow ≥ 3 L/min, and particle size ≤ 45 μm.

Furthermore, it is noteworthy that despite the use of fine particles (−45 μm), no significant particle agglomeration or ring formation was observed within the dynamic reactor. In conventional coal-based RKEF processes, ring formation is a critical issue often caused by the fusion of coal ash or the formation of low-melting liquid phases^23^. However, in this hydrogen-based reduction system, the absence of solid reductants eliminates ash-related accretion. Additionally, thermodynamic studies confirm that the predominant mineral phases formed at 900 °C, such as forsterite (Mg_2​_SiO_4_​) and enstatite (MgSiO_3_​), maintain a stable solid state well below their melting points^4^. This is consistent with findings by Satritama et al.^16^, who reported that saprolite ore retained its irregular particle morphology without agglomeration during hydrogen reduction up to 900 °C. Therefore, the hydrogen reduction process offers a distinct operational advantage by minimizing the risk of ring formation, even when processing fine feedstocks.

### Melting products after hydrogen reduction

**Fig. 8:**
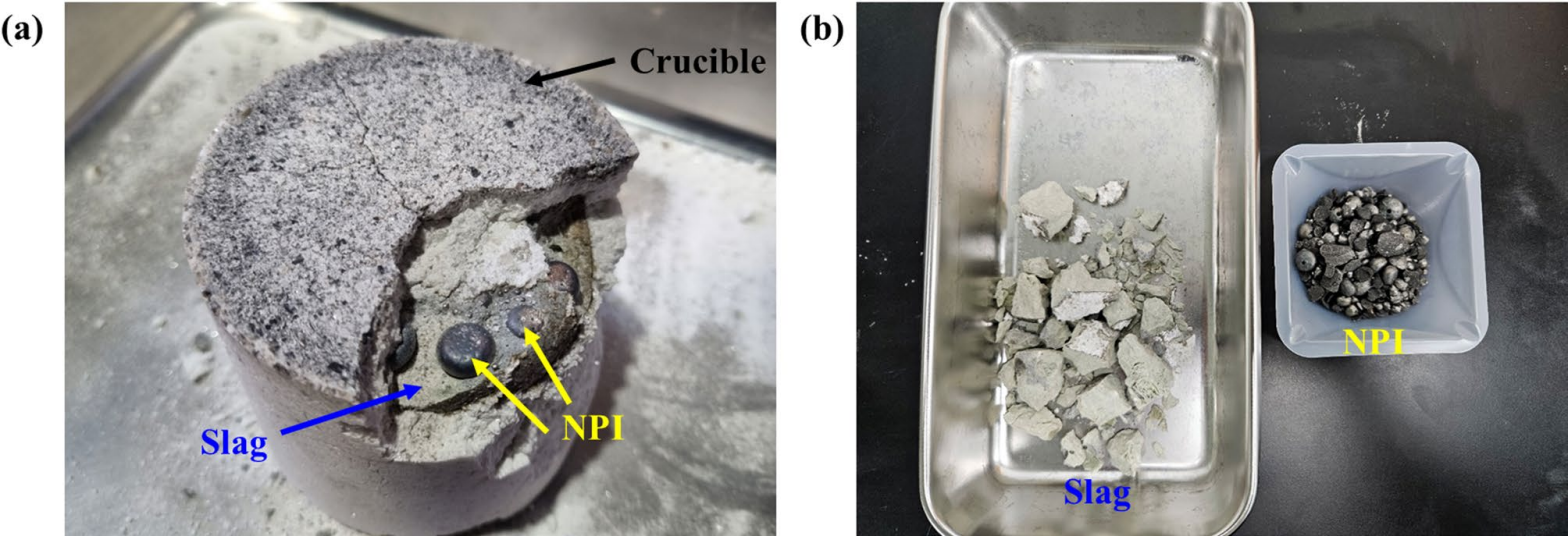
Melting products after heat treatment: (**a**) bottom of crucible; (**b**) NPI separation.

The samples recovered after the hydrogen reduction of the nickel oxide ore were melted in a vertical tube furnace (Fig. 2) to simulate the electric-furnace process. Ar gas was continuously supplied at 2 L/min throughout the thermal treatment to prevent oxidation. The melts were held at a maximum temperature of 1550 °C for 2 h to achieve complete melting and homogenization. After cooling to room temperature, the crucibles were disassembled, and the samples were retrieved. The results are shown in Fig. 8. The crucible detached readily from the sample, and the higher-density NPI was observed to accumulate at the bottom. The slag and NPI were completely separable via magnetic separation.

**Fig. 9:**
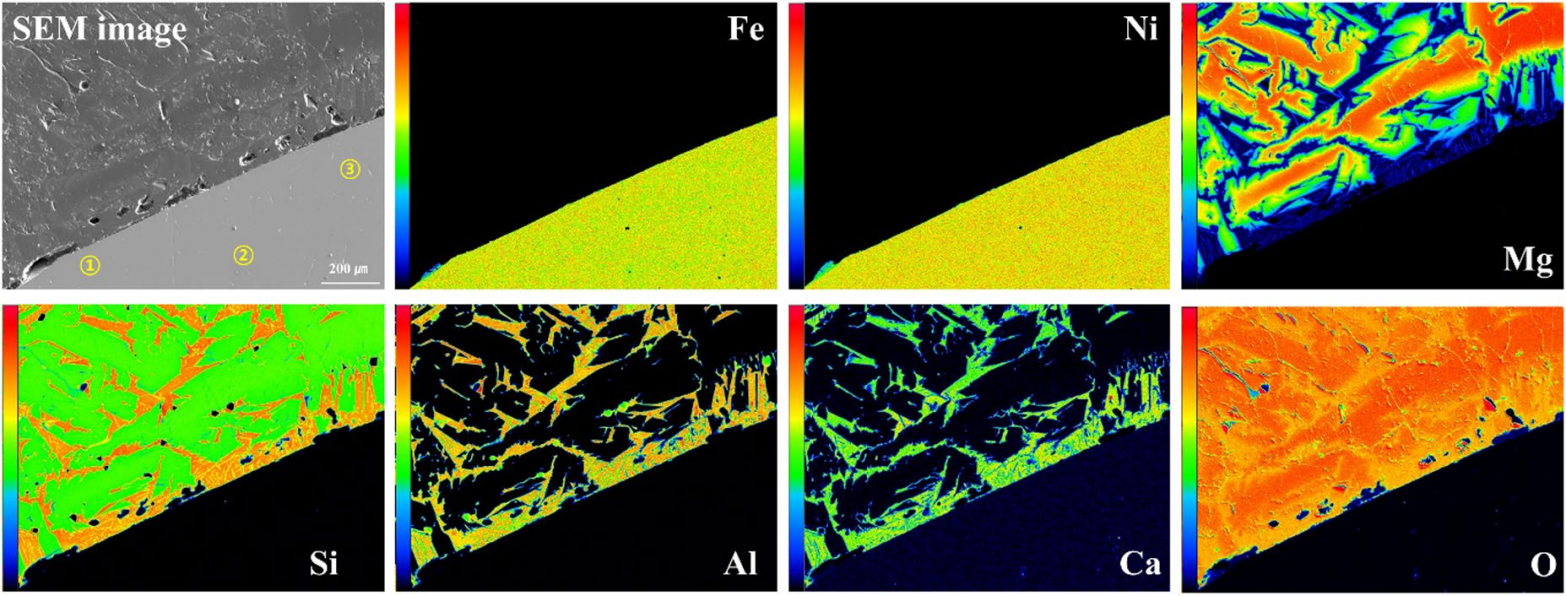
Melting products after heat treatment.

Figure 9 shows SEM images and the EPMA mapping of the cross section of the molten sample. The SEM images clearly distinguish between the upper slag region and the lower metal region, showing a distinct two-layer structure. The EPMA mapping results indicated that Fe and Ni were strongly distributed in the lower region, confirming the formation of NPI. WDS analysis was performed on three points to conduct a clear component analysis of the NPI, and the results are presented in Table 2. The Fe content in the NPI was found to be 73 wt% on average, and the nickel content was found to be 25 wt% on average. The upper region was dominated by Mg, Si, and O, and silicate crystals forming plate-like and needle-like structures were observed. This is consistent with the interpretation that Mg-rich silicates (mainly olivine/pyroxene-type, Mg₂SiO_4_, etc.) formed after the decomposition of serpentine constituted the slag matrix. Small amounts of Al and Ca were mainly confined to the upper silicate region and tended to be relatively concentrated at grain boundaries or around crystals. The low levels of Mg, Si, and O in the metal layer suggested that the mixing of oxides was limited, and the weak Fe and Ni signals in the silicate layer confirmed that phase separation was effectively achieved. This elemental separation pattern is consistent with the finding that NPI accumulates at the bottom after melting and can be completely separated from the upper slag through magnetic separation, as shown in Fig. 8. In conclusion, the EPMA mapping and WDS analysis results directly demonstrate that distinct phase separation between the Fe–Ni metal phase and the Mg-rich silicate slag was achieved during the melting stage, indicating that the pure hydrogen reduction-melting process is highly efficient for NPI recovery.

**Table 2:** Chemical composition of NPI determined via EPMA-WDS analysis. Sampling points No. 1, 2, and 3 correspond to the locations indicated in Fig. 9. N.D. Denotes “not detected.”.

No.	WDS (wt%)				
	Ni	Fe	Mg	Si	Cr
1	25.08	73.96	0.01	N.D.	0.03
2	25.07	73.21	N.D.	N.D.	0.04
3	25.01	72.94	N.D.	N.D.	0.01

## Conclusion

This study investigated the mineralogical characteristics of saprolite nickel ore and the effects of the process conditions and particle size on the reduction process under a pure hydrogen reduction atmosphere.

- Mineralogical analyses confirmed particle size-dependent variations in Fe, Mg, and Si, with higher Mg concentrations in the coarse fractions and higher Fe concentrations in the fine fractions, whereas nickel was uniformly distributed across all size fractions. These chemical differences are key factors influencing the reduction efficiency of saprolite ore.
- The reduction of saprolite nickel ore proceeded rapidly in a pure hydrogen atmosphere; with a reduction temperature of 900 °C, reduction time of 15 min, gas flow of ≥ 3 L/min, and particle size of ≤ − 45 μm, the reduction/mass loss (mass loss, wt%) reached ~ 20 wt% (mass loss), exhibited negligible time dependence, and was governed primarily by the gas flow rate and particle size.
- During melting at 1550 °C, clear separation was achieved between an Fe–Ni metallic phase (NPI; average Fe content ≈ 73 wt%, Ni content ≈ 25 wt%) and a Mg-rich silicate slag, enabling complete magnetic separation.
- Future work should focus on validating these findings in a continuous pilot-scale rotary kiln to assess long-term operational stability and evaluate the economic feasibility of hydrogen usage in industrial applications.

## Data Availability

The datasets generated and/or analyzed during the current study are available from the corresponding author on reasonable request.
